# Mapping and characterization of a novel powdery mildew resistance locus (PM2) in *Cannabis sativa* L.

**DOI:** 10.3389/fpls.2025.1543229

**Published:** 2025-03-13

**Authors:** Soren Seifi, Keegan M. Leckie, Ingrid Giles, Taylor O’Brien, John O. MacKenzie, Marco Todesco, Loren H. Rieseberg, Gregory J. Baute, Jose M. Celedon

**Affiliations:** ^1^ Breeding and Genetics Department, Aurora Cannabis, Inc., Comox, BC, Canada; ^2^ Department of Botany, University of British Columbia, Vancouver, BC, Canada

**Keywords:** *Cannabis sativa*, powdery mildew, disease resistance, genetic mapping, bulked-segregant analysis, marker assisted selection, plant breeding, sustainable agriculture

## Abstract

**Introduction:**

Breeding genetic resistance to economically important crop diseases is the most sustainable strategy for disease management and enhancing agricultural and horticultural productivity, particularly where the application of synthetic pesticides is prohibited. Powdery mildew disease, caused by the biotrophic fungal pathogen *Golovinomyces ambrosiae*, is one of the most prevalent threats to the cannabis and hemp industry worldwide.

**Methods:**

In this study, we used bulked-segregant analysis combined with high-throughput RNA sequencing (BSRSeq) to identify and map a novel single dominant resistance (R) locus (designated PM2), that strongly suppresses powdery mildew infection and sporulation in *Cannabis sativa*.

**Results and discussion:**

BSA mapped PM2 to chromosome 9. Histochemical analysis revealed that PM2-induced resistance is mediated by a highly localized hypersensitive response mainly in the epidermal cells of the host. Importantly, genetic markers capable of tracking PM2 resistance in breeding populations were developed using associated SNPs identified in this study. The ability to track PM2 will allow for successful introgression of PM resistance into elite cannabis cultivars and help move towards a more sustainable cannabis industry.

## 1 Introduction

*Cannabis sativa* L., commonly known as cannabis (marijuana or hemp) is a dioecious, diploid (2n=20), annual, flowering plant species belonging to the Cannabaceae family cultivated for its seed, oil, fiber, and bioactive compounds including cannabidiolic acid (CBDA) and tetrahydrocannabinolic acid (THCA) which have medicinal and psychoactive properties (Pate, 1983; Chandra et al., 2017; Radwan et al., 2017; Kumar et al., 2021). Following the legalization of medicinal and recreational uses of cannabis in many countries in the last few years, cannabis cultivation and related industries have seen a significant expansion (López-Ruiz et al., 2022). Plant diseases caused by fungal, bacterial, and viral pathogens are among the most important factors that threaten cannabis production (Punja, 2021). Powdery mildew (PM) is a widespread and economically damaging fungal disease affecting many indoor, greenhouse, and field grown cannabis crops around the world, including the cannabis industry in Canada and the USA (Pépin et al., 2021). PM disease in cannabis is mainly caused by the obligate biotrophic fungal pathogen *Golovinomyces ambrosiae*, previously known as *G. cichoracearum* (Pépin et al., 2018; Scott and Punja, 2020; Brochu et al., 2022). PM infection attacks the leaves, stems and flowers of cannabis, restricting photosynthesis and nutrient availability, causing premature leaf drop, poor flower quality and significant yield losses (Mihalyov and Garfinkel, 2021; Scott and Punja, 2020). The infection cycle in PM has three main phases: (i) conidium (spore) germination and epidermal penetration; (ii) mycelial network development on the host leaf; and (iii) conidia generation (asexual reproduction, also known as conidiation or sporulation) (Hückelhoven, 2005). Under optimal conditions, i.e. high humidity and moderate temperature, and access to susceptible cannabis cultivars, PM can complete its life cycle within 1-2 weeks post inoculation (wpi).

PM control in other crops is achieved by application of chemical fungicides, biological control agents, and agricultural practices; however, use of genetically resistant cultivars has historically been the most sustainable, effective, and economical approach in important crops such as wheat, barley and tomato (Agrios, 2005; Jørgensen and Wolfe, 1994; Seifi et al., 2014; Bapela et al., 2023). In countries with legal cannabis markets, use of agrochemicals and biological products are tightly controlled by regulatory agencies, and the few available products often exhibit limited protection (Mihalyov and Garfinkel, 2021; Scott and Punja, 2020). Therefore, developing commercial cultivars with genetic resistance to PM remains a highly valuable and sought-after goal in the cannabis industry (Sirangelo, 2023).

The plant immune system comprises several layers of constitutive and inducible defense mechanisms. Constitutive physical and biochemical barriers make up the outer layer of these defenses and can effectively suppress most pathogens. Nevertheless, those few pathogens that succeed in breaking through the preformed defenses will be dealt with by a complex defense machinery induced by the perception of the invading pathogen. Two main mechanisms are involved in the perception of an invading pathogen and the induction of an effective immune response: (i) recognition of pathogen-associated molecular patterns (PAMPs) by membrane-bound receptors leading to PAMP-triggered immunity (PTI), and (ii) detection of specific pathogen-derived effector proteins leading to effector-triggered immunity (ETI). Disease resistance (R) genes typically encode specific nucleotide-binding site leucine-rich repeat (NBS-LRR) proteins that can interact with and detect pathogen-derived effector proteins to induce an ETI response (Jones and Dangl, 2006; Hammond-Kosack and Kanyuka, 2007). Triggering of ETI causes the activation of defense-associated hormonal pathways, typically salicylic acid (SA), and several downstream genes coding for defense responses, such as the hypersensitive response (HR), production of antimicrobial pathogenesis related proteins (PR proteins), and cell wall fortifications, that ultimately deprive the pathogen of the plant’s nutritional resources (Hammond-Kosack and Kanyuka, 2007; Shamrai, 2022). R genes are often present in tandem arrays conferring vertical or qualitative resistance in the progeny through dominant Mendelian modes of inheritance where the effect of the dominant (resistance) allele can mask the effect of the recessive (susceptible) one. Qualitative resistance to PM has been reported in hops (*Humulus lupulus*), which is closely related to cannabis (Henning et al., 2017). The first report of a putative R gene mediated resistance against PM in cannabis (named PM1) suggested its location to be on chromosome 2 (Chr ID: NC_044375.1; GenBank acc. no. GCA_900626175.2) (Mihalyov and Garfinkel, 2021). Additionally, a mutation in the susceptibility (S) gene “Mildew Locus O” (*mlo*-mediated loss of susceptibility) has been reported to induce strong resistance to PM in cannabis (Stack et al., 2024).

Bulked-segregant analysis (BSA) coupled with high throughput sequencing has become a popular method for quantitative trait locus (QTL) mapping and is widely used for mapping disease resistance loci within economically important crops (Takagi et al., 2013; Win et al., 2017; Imerovski et al., 2019; Shen et al., 2019; Liang et al., 2020), including those effected by powdery mildew (Ma et al., 2021; Cao et al., 2021). BSA involves the creation of a bi-parental segregating population where both parents display opposing phenotypes for a quantitative or qualitative trait of interest. Unlike traditional QTL mapping where all individuals in a segregating population are genotyped, BSA involves selecting individuals with opposing values for qualitative traits, or individuals from both tails of the distribution for quantitative traits. Selected individuals are grouped into two bulks, representing the extremes of the target trait. This makes BSA attractive for mapping disease resistance QTLs as bulks can be easily made from resistant and susceptible plants. Genotyping in BSA is limited to the two bulks of plants, drastically reducing sequencing cost. Furthermore, several methods of BSA have been developed to utilize single nucleotide polymorphisms (SNPs) called from RNA-Seq data, referred to as BSR-Seq, which provides the required read depth and gene expression data for a lower cost compared to DNA sequencing (Liu et al., 2012; Hill et al., 2013).

Herein, we report the discovery of a novel single dominant PM resistant locus (PM2) that confers strong resistance to PM disease in cannabis. We present evidence indicating that PM2 acts through the induction of a highly localized ROS accumulation in the epidermal and mesophyll layers of the host leaf tissue resulting in HR, the arrest of the pathogen growth, and suppression of its sporulation. We identified two genotypes containing PM2 within a large cannabis diversity population screened for PM resistance. Using BSR-Seq, we mapped PM2 to chromosome 9 (Chr ID: NC_083609.1, Pink Pepper reference genome) and developed genetic markers that can be used to introgress PM2 resistance into elite cannabis commercial cultivars.

## 2 Materials and methods

### 2.1 Plant material

A large diversity population of 510 genotypes called CanD (Cannabis Diversity), including the parental lines W03, N88, and AC, is part of Aurora’s germplasm and is maintained as mother plants to provided clones for experiments. The W03xAC and N88xAC F_1_ mapping populations were generated form a PM-susceptible (AC) parent and PM-resistant (W03 and N88) parents, all described as female, type-I (THC-dominant) drug-type cannabis. Selfed (S_1_) progeny were generated by applying a foliar spray of silver thiosulfate (Millipore Sigma) at a concentration of 0.02M to clones from W03 and N88 parental lines on days 1 and 8 of a short-day photoperiod treatment to induce male flower formation and self-pollination (Mohan Ram and Sett, 1982).

### 2.2 PM infection assays, disease index, and plant growth conditions

All CanD genotypes were evaluated for PM susceptibility using a clone assay, following a completely randomized design (CRD) with at least 6 replicates per genotype. Plantlets were rooted for 14 days in rockwool cubes and inoculated 3 weeks after cloning. Inoculation of healthy plantlets were carried out through “dusting”, whereby fungal spores from a sporulating infected leaf are transferred by tapping the leaf and depositing fresh spores onto the surface of a non-infected leaf. PM spores were sourced from highly infected plants kept in isolation. Disease evaluation and scoring was performed at 4 wpi. Similar methods of inoculation and disease evaluation were used for seedling infection trials. To mimic a production cycle from cloning to harvest, an adult plant assay was performed over the course of a 12-week period. In all assays, plants were grown at 23°C and 80% relative humidity (RH) for the first 48 h (to ensure high levels of spore germination), and then kept at 70% RH during the rest of the infection trial. Photoperiod was 16 h of light and 8 h of dark for clonal propagation and rooting, vegetative growth during the clone assay, and the first two weeks of the adult plant assay. After 2 weeks of growth under vegetative conditions, photoperiod was changed to 12 h of light and 12 h of dark for 10 weeks to induce flowering in the adult plant assay.

Disease severity was assessed with a “disease index” following a method previously developed (Seifi et al., 2013a, 2021), using qualitative severity scores from zero to four according to the area covered by colonies with PM sporulation (“0”, healthy leaves with no signs of infection; “1”, less than 25% coverage; “2”, 25-50% coverage; “3”, 50-75% coverage; and “4”, 75-100% coverage). Disease index was calculated for each genotype by scoring the disease severity in the three most infected leaves from the three most infected plants per genotype using the following formula:


$$DI=\frac{\left(0\times n_{0}\right)+\left(1\times n_{1}\right)+\left(2\times n_{2}\right)+\left(3\times n_{3}\right)+\left(4\times n_{4}\right)}{N\times 4}\times 100$$

where DI shows the disease severity in percentage; $n_{0},n_{1},n_{2},n_{3},n_{4}$ are the number of leaves with disease severity scores of 0, 1, 2, 3 and 4 respectively; and N is the total number of leaves evaluated. Disease severity was scored at 4 wpi for clone and seedling assays, and at 12 wpi for adult plant assay.

### 2.3 Microscopy

The accumulation of hydrogen peroxide was visualized using 3,3-diaminobenzidine (DAB) staining following the protocol devised by Thordal-Christensen et al. (1997). Briefly, samples were treated with DAB-HCl (1 mg/ml) for 3 hours before being fixed in 100% ethanol for downstream microscopy. Fungal structures were stained with 0.1% trypan blue in 10% acetic acid following the protocol described by Seifi et al. (2013a). All staining protocols were followed by extensive rinsing steps in demineralized water and samples were subsequently mounted in 50% glycerol before brightfield microscopic observations using an Olympus (Tokyo, Japan) CX443 microscope.

### 2.4 RNA isolation

W03xAC and N88xAC F_1_ mapping populations were grown from seed in greenhouse conditions under 18 hours of light. At 3 weeks, plants were manually infected with PM by dusting fresh spores as described in the clone infection assay. Evaluations and tissue sampling were conducted at week 7 (4 wpi). For each F_1_ population, 1 g of leaf sample per plant from 25 resistant and 25 susceptible plants were collected and flash frozen in a slurry of isopropanol and dry ice. Bulks of frozen leaf samples from each population were separately ground in a mortar with liquid nitrogen and 200 mg of ground material was used for RNA isolation using the PureLink^™^ Plant RNA Reagent (ThermoFischer Sci.) following the manufacturer small scale protocol. RNA quality and concentration were assessed using an Agilent Technologies 2100 Bioanalyzer. Only samples with high quality RNA (RNA integrity numbers > 7) were used for RNA sequencing.

### 2.5 RNA-Seq

Construction of mRNA libraries and sequencing was performed at SBME-Seq center at the School of Biomedical Engineering, University of British Columbia. Each library was sequenced to a depth of ~20 million PE-reads (150 bp long), using an Illumina NextSeq2000. Library quality and presence of adaptors in raw RNA-Seq reads were analyzed using FastQC (Andrews, 2010). Skewer (Jiang et al., 2014) was used to trim reads to a Phred score no less than Q28. STAR (version 2.7.11a, Dobin et al., 2013) was used to map processed RNA-Seq reads to the Pink Pepper (GenBank acc. no. GCA_029168945.1; Ryu et al., 2024) and CBDRx (GenBank acc. no. GCA_900626175.2; Grassa et al., 2021) reference genomes. Mapped RNA-Seq reads were assembled into transcripts and quantified for gene expression using Stringtie v2.1.4 (Kovaka et al., 2019). To calculate the relative expression difference between susceptible and resistant bulks for PM2 gene candidates, the mean gene expression between the W03xAC and N88xAC populations was used. Gene expression was measured in Transcripts per Million (TPM), reported by Stringtie. Relative expression difference between bulks for PM2 candidate genes was calculated as:


$$RED=\;\frac{TPM_{Resistant\;Bulk}\;-\;TPM_{Susceptible\;Bulk}}{TPM_{Resistant\;Bulk}}$$

RED is the relative expression difference, where TPM_Resistant Bulk_ and TPM_Susceptible Bulk_ are the average gene expression between both W03xAC and N88xAC population for the resistant and susceptible bulks respectively.

### 2.6 Variant calling

SNP calling was performed by following The Broad Institute’s best practices for RNA-Seq short variant discovery (SNPs + Indels) using GATK (versions 4.3, McKenna et al., 2010). GATK’s Picard implemented MarkDuplicates and SplitNCigarReads tools were used for marking read duplicates and splitting intron spanning reads, respectively. Read duplicates were only marked for aiding variant calling and not removed. GATK’s HaplotypeCaller was used to call variants in each bulk. Resulting variants that did not meet the following quality statistics were removed: quality (QUAL) > 30, quality by depth (QD) > 2.0, Strand Odds Ratio (SOR)< 3.0, FisherStrand (FS)< 60.0, RMS Mapping Quality (MQ) > 40.0, Mapping Quality Rank Sum Test (MQRankSum) > -12.5, and Read Position Rank Sum Test (ReadPosRankSum) > -8.0. Lastly, only bi-allelic (SNPs) variants were selected for BSR-Seq analysis.

### 2.7 Bulked segregant RNA-Seq

BSR-Seq was conducted using a custom in-house R program written to implement both the Euclidean distance metric developed by Hill et al. (2013) and the Bayesian statistical approach developed by Liu et al. (2012). Both methods rely on allele read counts from both susceptible and resistant bulks to determine genetic linkage between markers and the causal gene of a trait of interest. SNPs common to both bulks (susceptible and resistant) were used for analysis. SNPs called from read counts of less than 20 reads were filtered out as their allelic frequencies cannot be accurately measured. The empirical Bayesian approach by Liu et al. (2012) estimates the conditional probability of no recombination taking place between a SNP and the causal gene in the resistance bulk, given the allelic read counts of each SNP. We incorporated the R script written by Liu et al. (2012) that implements the Bayesian approach into our pipeline, adjusting two parameters: the number of plants per bulk, 25, and the total genetic length, which for Cannabis has been reported to be 818cM (Grassa et al., 2021). The Euclidean distance method was implemented as described by Hill et al. (2013) using the following formula:


$$\begin{array}{c} ED=\\ \;\sqrt{{\left(A_{Res}-\;A_{Sus}\right)}^{2}+{\left(C_{Res}-\;C_{Sus}\right)}^{2}+{\left(G_{Res}-\;G_{Sus}\right)}^{2}+{\left(T_{Res}-\;T_{Sus}\right)}^{2}} \end{array}$$

where each letter represents the allelic frequency of its corresponding nucleotide, with subscripts *Res* and *Sus* indicating the phenotypic pool, resistant and susceptible respectively.

### 2.8 Candidate gene selection

Candidate genes identified within the PM2 QTL region were selected based on the following criteria. Genes with annotations in the Pink Pepper reference genome known to be involved in disease response in other species were identified and selected for further analysis. To confirm the quality of gene annotations in the region of interest, we took the CDS sequence from genes having annotations related to disease response and blast them to Arabidopsis protein sequences to assess the quality of their predicted annotation. Furthermore, we searched and selected genes with conserved leucine-rich repeats (LRR) domains as probable gene candidates due to LRRs known roles in characterized R-genes. Finaly, an extensive literature review was conducted to identify homologs of candidate genes that had been shown to play a role in disease response in other plant species.

### 2.9 DNA isolation

Single leaflets were collected from 10-14 day old seedlings into 96-well deepwell plates containing 2mm borosilicate beads. Leaflets were lyophilized overnight, then ground into powder using a QIAGEN TissueLyser II. DNA purification was done following standard extraction protocol with a sbeadex Mini Plant DNA Purification Kit^™^ (Biosearch Technologies) on an Oktopure Liquid Handling system.

### 2.10 Genotyping

Genotyping was done using PCR Allelic Competitive Extension (PACE) 2.0 chemistry (3CR Bioscience, Essex, UK). Assays were designed by the 3CR Bioscience assay design service. All PACE reactions were performed on a QuantStudio 7 (Applied Biosystems) using the manufacturer suggested PACE reaction volumes and cycling conditions. PACE primer sequences are provided in **Supplementary Table S1**.

## 3 Results

### 3.1 Germplasm screening for PM resistance

**Figure 1:**
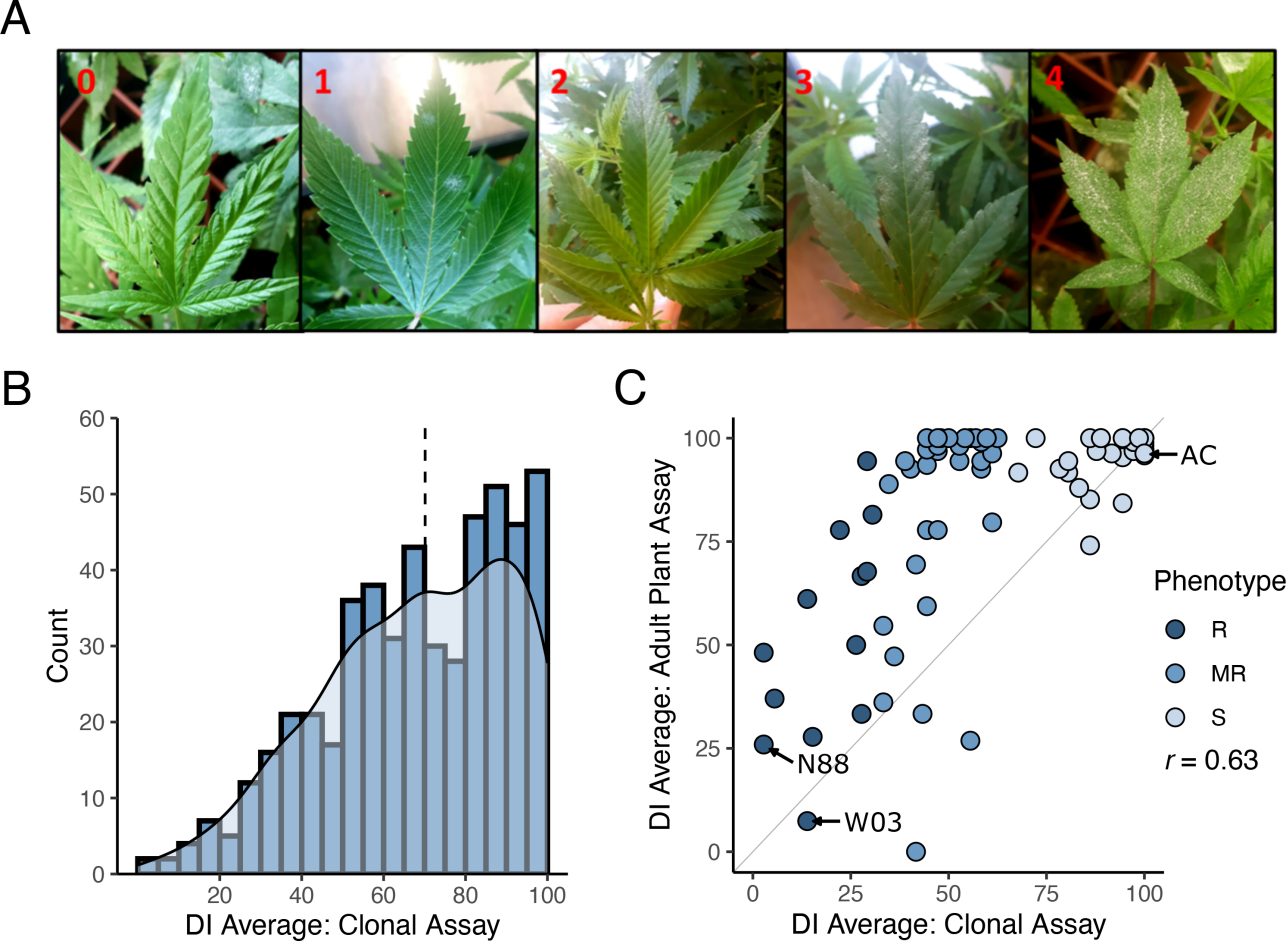
Powdery mildew disease index on cannabis CanD diversity population. **(A)** Illustration of the severity scale of PM infection on cannabis leaves used for disease index evaluation. 0: no symptoms; 1: less than 25% PM coverage (few restricted, not spreading colonies); 2: PM covering 25-50% of the leaf; 3: PM covering 50-75% of the leaf; 4: PM covering 75-100% of the leaf surface. **(B)** Distribution of DI results from clonal infection assay. Blue bars indicate number of genotypes within a defined DI range (histogram), while the light blue shaded area depicts kernel density estimate of the DI distribution. Black dashed line denotes the median of the distribution. **(C)** DI results from the adult plant infection assay of 90 genotypes selected from the CanD population compared to their corresponding clonal infection assay results. Genotypes from all three resistance categories were selected: Susceptible (S) shown in light blue, moderately resistance (MR) shown in blue, and resistant (R) shown in dark blue. The diagonal line (x=y) is shown in light grey as reference. The Pearson correlation between adult infection assay versus clonal infection assay was *r* = 0.63.

To identify new sources of PM resistance, we screened a total of 510 genotypes in our cannabis germplasm collection, including production cultivars, landraces, and exotic lines, and called this population CanD for **Can** nabis **D** iversity. All 510 genotypes were initially evaluated for resistance/susceptibility to PM using a disease index (DI) measured using a clone infection assay (**Figure 1A**, see Materials and Methods). The observed distribution of DI in the CanD population was left-skewed, with more than 70% of the genotypes scoring a DI > 50 indicating a high prevalence of PM susceptibility (**Figure 1B**). The continuous nature of the distribution of DI in the CanD population suggests that multiple loci, most with small effects, contribute to PM resistance in cannabis.

To confirm if the resistance to PM observed in clones would persist through the flowering phase of the plant’s life cycle, we performed an adult plant assay on a subset of 90 CanD genotypes. Genotypes were selected to represent the following three phenotypic groups based on their clonal infection assay score: (i) resistant (R: DI ≤ 33), (ii) moderately resistant (MR: 33< DI< 66), and (iii) susceptible (S: 66< DI ≤ 100). All resistant genotypes identified in the CanD population were included in the adult plant assay to confirm the resistance to PM observed in clones. Disease pressure in the adult-plant experiment was significantly higher than in the clone assay due to the longer duration (12 vs. 4 weeks) and repeated exposure to high inoculum levels and fresh spores. In the adult plant assay, many genotypes showed an increase in PM susceptibility compared to the clonal assay (**Figure 1C**, genotypes above the plot diagonal). In severe susceptibility cases, sugar leaves of the flowers, petioles, and parts of the stem tissue were also colonized by the PM pathogen. Overall, the DI in the adult plant assay positively correlated with the results of the clonal assay for the 90 genotypes tested in both experiments (*r* = 0.63). A final group of 12 resistant genotypes (DI< 50) were selected from the adult-plant infection trial. Within this group, 5 genotypes showed strong resistance responses to PM (DI< 33), despite the high disease pressure and length of the assay.

### 3.2 Mode of inheritance of the observed R locus

**Figure 2:**
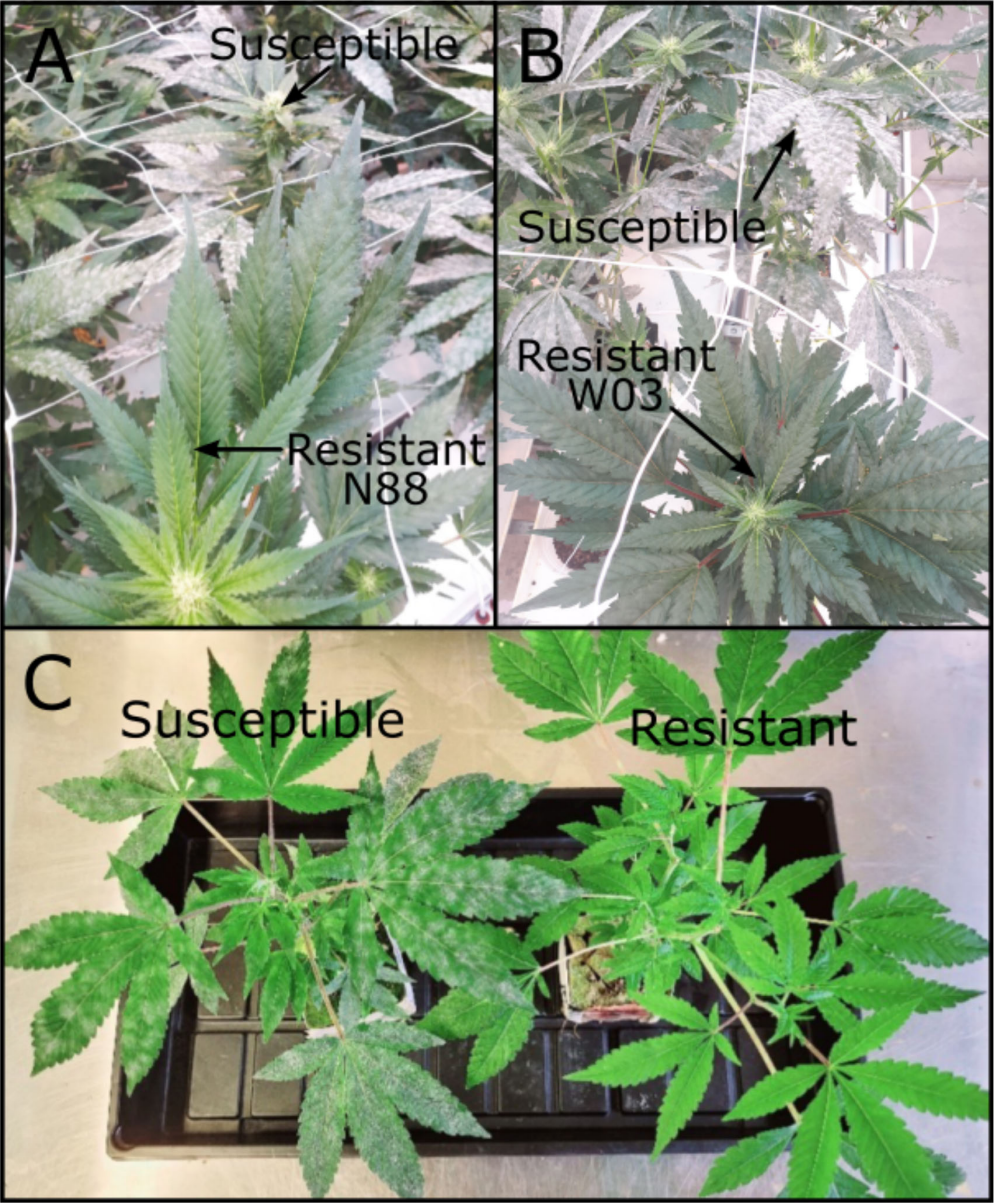
PM resistant genotypes N88 and W03. **(A)** N88 (bottom) next to a PM infected susceptible genotype (top) at 10 wpi during adult plant infection trial. **(B)** W03 (bottom) next to PM infected susceptible genotypes (top) at 10 wpi during adult infection trial. **(C)** Resistant W03xAC-derived F_1_ progeny (right) next to a PM infected susceptible sibling (left) at 4 wpi.

Twelve different F_1_ populations derived from highly resistant (R) and susceptible (S) genotypes were subjected to infection trials using the clone assay to determine the mode of inheritance of PM resistance (Materials and Methods). Among the populations tested, two exhibited a 1:1 ratio for R:S in their F_1_ progeny (**Figure 2**), indicating that resistance to PM in these cases is mediated by a single dominant locus R gene, and that the resistant parents are heterozygous for that locus. The identified resistant parents, W03 and N88 were crossed with a susceptible cultivar, AC (**Table 1**). The progeny in the remaining nine F_1_ populations tested did not show strong PM resistance, suggesting a multigenic origin of pathogen resistance in the parents of the crosses.

**Table 1:** Chi-squared tests for goodness of fit for the two F_1_ test cross populations.

Cross (R × S)	Resistant-progeny Exp. vs. Obs.		Susceptible-progeny Exp. vs. Obs.		Chi-square test		
					χ2	df	P-value
N88 × AC	33.5	36	33.5	31	0.373	1	0.5413
W03 × AC	22.5	24	22.5	21	.200	1	0.6547

### 3.3 QTL mapping using bulked segregant RNA-seq analysis

To map the observed dominant PM resistance in the cannabis genome, we performed bulked segregant analysis on the two previously described bi-parental F_1_ populations made from PM-resistant and PM-susceptible genotypes (N88xAC and W03xAC). For each F_1_ population, susceptible and resistant bulks were created consisting of 25 plants each grouped by their respective phenotype (PM susceptible and PM resistant). RNA sequencing and reference genome alignment was performed on each bulk (Materials and Methods). For the W03xAC population, bulks resulted in 25,150,091 and 17,017,509 reads uniquely aligned to the Pink Pepper Cannabis reference genome for resistant and susceptible bulks respectively and used for SNP calling. A total of 65,202 SNPs shared between both bulks were used for BSR-Seq after filtering for quality scores and read depth (see Materials and Methods). Similarly, the N88xAC population yielded 24,179,503 and 21,576,849 uniquely mapped reads in the resistant and susceptible bulks respectively, and 82,099 shared SNPs after filtering.

**Figure 3:**
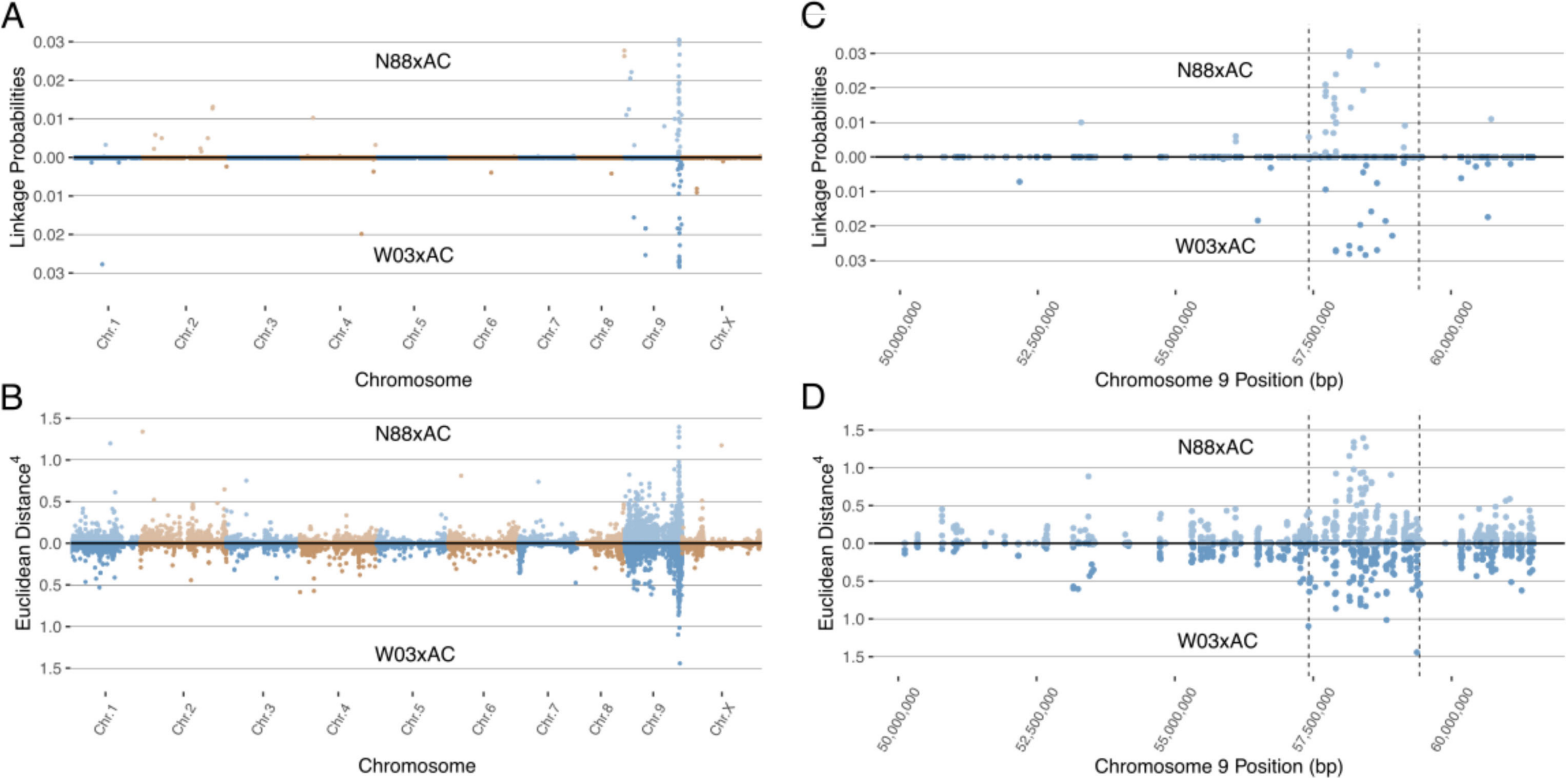
Mapping the PM2 locus using BSR-Seq in both N88xAC (top half) and W03xAC (bottom half) F_1_ segregating populations. Pink Pepper genome was used as reference (GenBank assembly GCA_029168945.1). **(A)** BSR-Seq mapping using the Bayesian implementation to estimate the probability of SNPs linked to PM2 resistance. **(B)** BSR-Seq mapping using the Euclidean distance (ED) metric. ED values for each SNP have been raised to the 4^th^ power to increase signal to noise ratio. Alternating colours denote chromosomes in **(A, B)**. Both Bayesian **(C)** and ED **(D)** metrics identify a region on chromosome 9 between 57,417,178bp and 59,418,457bp containing SNPs associated with PM2 resistance. Vertical dashed lines denote the boundaries of the PM2 associated region, defined by increased Bayesian probability and ED scores.

To identify the region associated with PM resistance in these populations, we used two separate methods developed for BSR-Seq: the Bayesian implementation by Liu et al. (2012) to estimate the probability of SNPs in linkage disequilibrium with the casual gene of a trait of interest, and the Euclidean distance metric developed by Hill et al. (2013), a robust measure of allele frequency differences between bulks. The Bayesian approach identified a cluster of SNPs with high probability of being linked to PM resistance on chromosomes 9 in both the W03xAC and N88xAC populations (**Figure 3A**). Using the Euclidean distance metric, a strong signal was observed in the same region of chromosome 9 in both populations (**Figure 3B**). Both the ED and Bayesian probability metrics identify an overlapping region of approximately 2.0Mbp. The boundaries of this region were defined between positions 57,417,178 and 59,418,457 based on SNPs with elevated ED scores and increased posterior probabilities to being linked with PM resistance (**Figures 3C, D**). To further confirm that the identified region is associated with PM resistance, we repeated the BSR-Seq analysis on a second publicly available reference genome, CBDRx. Using both the Bayesian probability and ED metrics, a cluster of SNPs highly associated with PM resistance was observed on chromosome 9 (NC_044376.1) corresponding to the same region observed in the Pink Pepper reference genome (**Supplementary Figure S1**). These results strongly suggest that both F_1_ populations are segregating for the same single dominant PM resistance locus, hereafter named PM2.

### 3.4 Development and validation of genetic markers for breeding PM2 resistance

**Figure 4:**
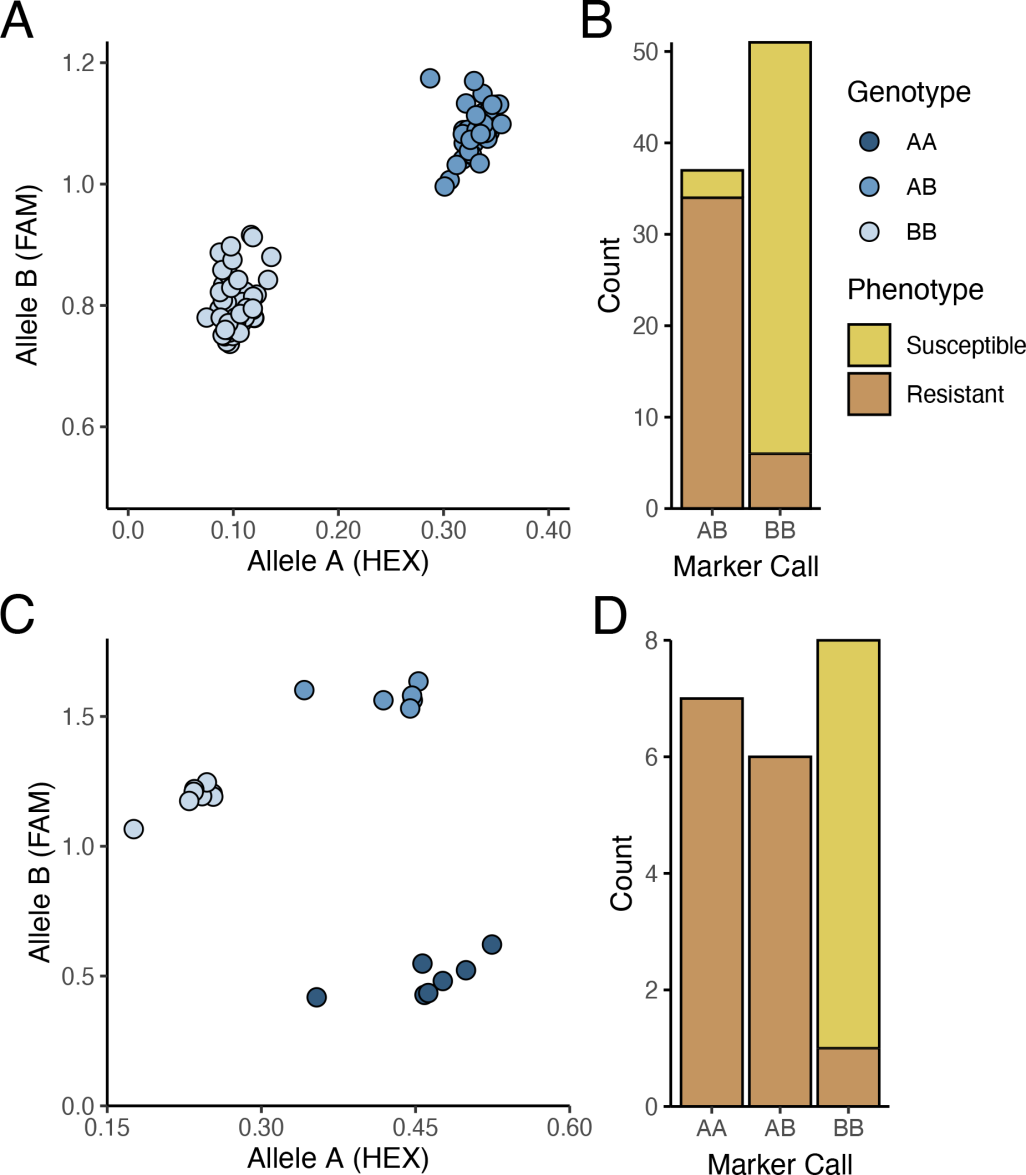
PACE results for marker MR110 tracking PM2 mediated resistance. **(A)** Segregation of AB (resistant) and BB (susceptible) alleles in W03xAC F_1_ genotypes. Results depict 1 of 2 independent qPCR runs testing marker MR110. **(B)** Accuracy of marker MR110 on the F_1_ population from **(A)**. Of the 37 F_1_ progeny genotyped as AB, 34 plants were resistant to PM (91.9%). Fifty-one F_1_ progeny were genotyped as BB, with 45 of them susceptible to PM (88.2%). **(C)** Segregation of AA (resistant), AB (resistant), and BB (susceptible) alleles in W03 S_1_ population. **(D)** Accuracy of marker MR110 on the S_1_ population from **(C)**. Of the 7 S_1_ plants genotyped as AA and 6 S_1_ genotyped as AB, all were resistant to PM. Eight S_1_ plants were genotyped as BB with all but 1 plant being susceptible to PM (87.5%).

The PM2 QTL defined region contains 2,342 SNPs for the W03xAC population and 2,208 SNPs for the N88xAC population. We developed PACE genotyping assays to validate five SNPs as genetic markers for PM2 resistance in W03xAC and N88xAC F_1_, and W03 and N88 selfed (S_1_) populations (**Supplementary Table S2**). The accuracy of the markers was tested by comparing their genotype calls to the populations phenotypes as determined by clonal-infection assay. For the W03xAC and N88xAC F_1_ populations, markers had a prediction accuracy between 92 and 99% (**Table 2**). Marker accuracies were slightly lower when tested on W03 and N88 selfed populations, ranging between 86-93%. The number of genotyped plants in the S_1_ populations were however smaller compared to the number tested in the F_1_ populations (**Table 2**). To evaluate allelic segregation in the S_1_ populations, a chi-squared test for deviation from Mendelian ratios of inheritance was used and showed that all populations fell within accepted ranges (**Table 3**). Marker 110 was extensively tested in both F_1_ and S_1_ populations, with its genotype calls and marker accuracy depicted in **Figure 4**.

**Table 2:** Marker accuracy results and flanking sequence.

Marker Assay	Flanking Sequence	Validated population	Marker accuracy
MR110	ATCACCAGCCAAGAATCCCACCACWGGAACCACATCCTTAAC CTCTCCAAGCCTCCCCATGGGGCACTCATCCACCACTTTCTTC AAGTCATCCTCACT**[C/T]**TTCCCCGAAAAGAACATATCSGTCGCGATTGGTCCCGGKGCCA MGCAGTTGGCCGTGATCCCCRTCCCCTTYAGCTCCTTAGCAAGTATCTTGATCATCG	W03xAC	95% (n=216)
		N88xAC	98% (n=187)
		W03 S_1_	90% (n=21)
		N88 S_1_	86% (n=28)
MR121	ATACTATGATGGCCGTGAAAATGTCCACACAGATTATCCTGTCGCGGATCTGTTG CAGATGATGGGTCATGCTAGTCGGCCATTGCTAGATAATTCCGG**[T/G]**AAATGTGT GATCCTCTGCCATGCACCTCGTAAAGAATATTACAAGGAGTTCT TATATGAATCATTCCCAGTTGAAAGCCATTTACACCACTACTTGCATG	W03xAC	92% (n=176)
		W03 S_1_	90% (n=21)
MR131	GAGAAAGCACAGTGAGACTGTGGTGGTGGCACCAGTGAAGAAGGATGATACAGCTGC AGAGAGGCCTMAAAGGACWCTTTTGKGTTGGAAAGAGAAGAA**[G/A]**AATGAAGCAGAMSCAGAAACTGAATCAACTCCGTTTTTCAGGAACAAAGAGAAGRTTT TRGTTACTTGTTCTCGAAGAATTAATTACAGGTTTGWAAAAA	N88xAC	99% (n=171)
		N88 S_1_	93% (n=28)
MR124	TGAGACTGTGGTGGTGGCACCAGTGAAGAAGGATGATACAGCTGCAGAGAGGCCTMAA AGGACWCTTTTGKGTTGGAAAGAGAAGAARAATGAAGCAGA**[A/C]**SCAGAAACTGAATCAACTCCGTTTTTCAGGAACAAAGAGAAGRTTTTRGTTACTTGT TCTCGAAGAATTAATTACAGGTTTGWAAAAAKAATCRATCAAA	N88xAC	97% (n=184)
		N88 S_1_	93% (n=28)
MR125	TGTGAGAAAATTCARAAAAATGACACGGTTRAAAGGATAAGAASTCGGAAAAGAAAC AATACCCARTCCAAGATCAACAAGATATAGTTTCTTCTCATC**[G/A]**ACTGTTCCAGGTTGACCAAGTAAAAAATTCTCGGGCTTTACATCTCCATGCAC AAACCTATTTAGAATTAGAAAATTTGAAAATAATCAACAATGCAWTT	N88xAC	99% (n=180)
		N88 S_1_	93% (n=28)

Marker accuracy for F_1_ populations are the combined results from two independent qPCR runs for each population, resulting in the higher total N value compared to S_1_ populations, which were single runs per populations. Bold letters within brackets indicate the polymorphic site of the KASP markers with allelic variants.

**Table 3:** Chi-squared test for deviation from mendelian patterns of inheritance (df=2).

Marker	Population	AA		AB		BB		χ^2^	p-value
		O	E	O	E	O	E		
MR 110	W03 S_1_	7	5.25	6	10.5	8	5.25	3.9524	0.1386
MR 110	N88 S_1_	8	7	12	14	8	7	0.5714	0.7515
MR 121	W03 S_1_	8	5.25	6	10.5	7	5.25	3.9524	0.1386
MR 124	N88 S_1_	8	7	11	14	9	7	1.3571	0.5074
MR 125	N88 S_1_	8	7	12	14	8	7	0.5714	0.7515
MR 131	N88 S_1_	8	7	11	14	9	7	1.3571	0.5074

O, observed, E, expected.

### 3.5 Putative candidate genes at the PM2 locus

**Figure 5:**
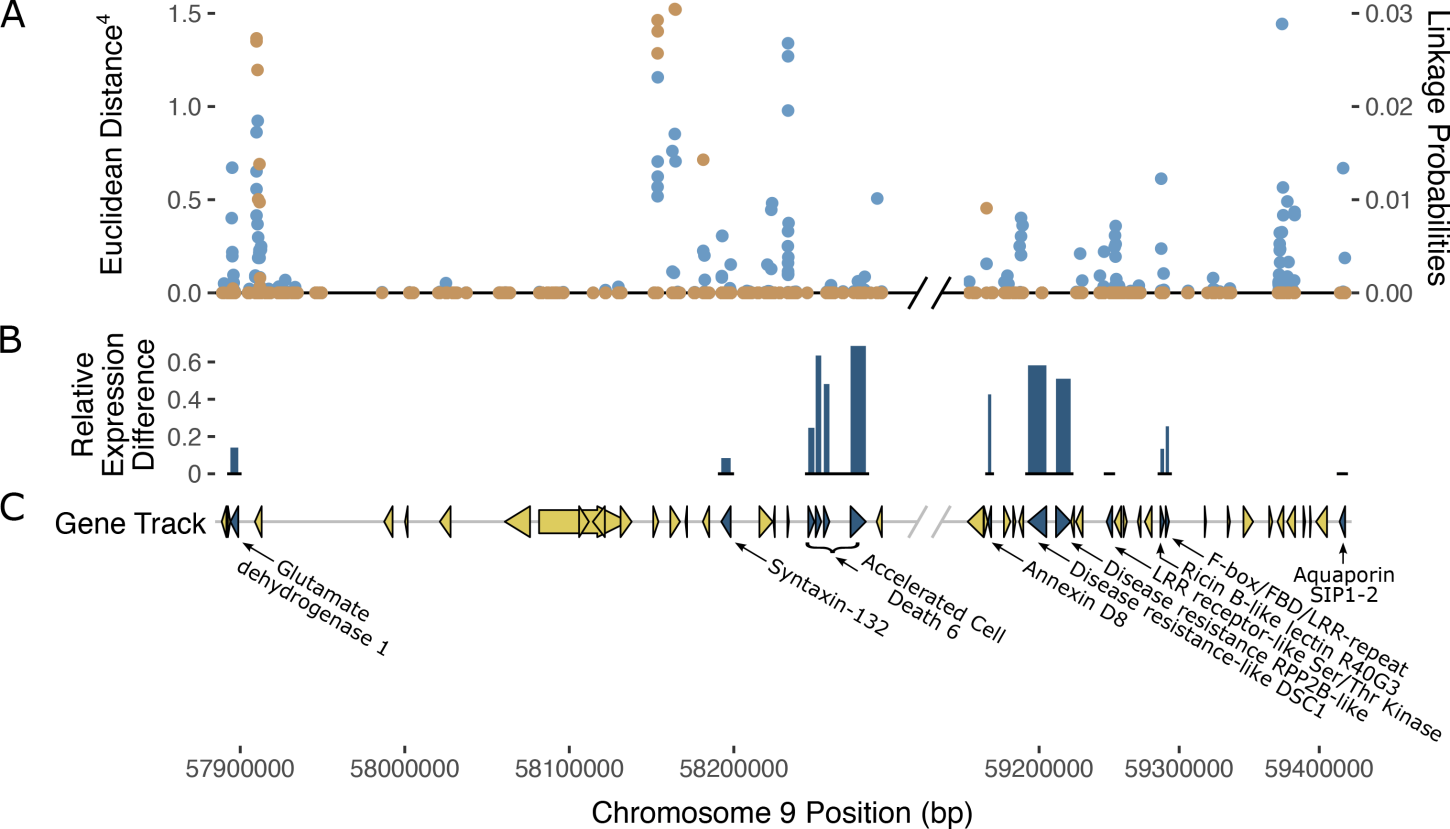
Possible gene candidates mediating PM2 resistance. **(A)** SNPs within the PM2 QTL defined region and their corresponding ED scores (left axis) are shown in blue (top track). Superimposed in orange are the Bayesian SNP linkage probabilities (right axis). SNP ED and Bayesian probabilities are the combined datasets from both N88xAC and W03xAC F_1_ populations. **(B)** Relative expression difference between bulks. Height of dark blue bars (middle track) indicate the relative level of gene expression difference between resistant and susceptible bulks for gene candidates. Relative expression difference for each gene was calculated as: (Resistance – Susceptible)/Resistance. Expression was measured in transcripts per million (TPM). **(C)** Gene track and annotations within PM2 QTL region. Gene markers (triangles and arrows) in dark blue denote genes that may be responsible for PM2 resistance based on predicted annotation and previous studies in other plant species. Yellow gene markers denote genes less likely to be responsible for PM2 resistance. Non-protein coding genes are not displayed in the gene track. Diagonal black and grey lines denote a break in chromosome position excluding a region where genes had annotations unrelated to defense responses against pathogens.

Analysis of gene annotations in the PM2 QTL region revealed 13 coding sequences with known functions in disease resistance in other plant species which represent putative candidate genes that could explain PM2 resistance (**Figure 5**; **Table 4**). The ratio of expression between resistant and susceptible bulks in some of the selected genes indicated differences that provide additional evidence supporting a putative role in PM2 resistance (**Supplementary Table S3**).

**Table 4:** Putative candidate genes within the PM2 region associated with defense signaling and pathogen response.

Gene name	Start (bp)	End (bp)	Annotation	Mechanism	Pathosystem	Reference
LOC115722251	57894037	57898806	glutamate dehydrogenase 1	TCA cycle exhaustion - cell death	Tobacco - response to biotrophic pathogens	Seifi et al., 2013b
LOC115722780	58192366	58198073	syntaxin-132	SA defense pathway - PR1 expression	Tomato *- Oidium lycopersicum* (powdery mildew)	Bracuto et al., 2017
LOC115723470 LOC133031067 LOC133031066 LOC133031533	58249744 58270938 58245244 58254683	58253111 58280233 58248979 58258108	ACCELERATED CELL DEATH 6 ACCELERATED CELL DEATH 6 ACCELERATED CELL DEATH 6 ACCELERATED CELL DEATH 6	SA defense signaling, cell death SA defense signaling, cell death SA defense signaling, cell death SA defense signaling, cell death	Arabidopsis - response to PM (*G. cichoracearum*) and *Pseudomonas syringae*	Dong, 2004; Todesco et al., 2010
LOC115723381	59163552	59165815	annexin D8	ROS accumulation, callose formation	Wheat - *puccinia striiformis* (yellow rust)	Shi et al., 2023
LOC133030743	59192095	59205193	disease resistance-like protein DSC1	Potential R gene	Arabidopsis - root-knot nematode	Warmerdam et al., 2020
LOC133030744	59212063	59222527	disease resistance protein RPP2B-like	Potential R gene	Arabidopsis - *Hyaloperonospora arabidopsidis* (downy mildew)	Sinapidou et al., 2004
LOC115722098	59248198	59252239	putative LRR receptor-like protein kinase At2g24130	Potential R gene		
LOC115722788	59286614	59289221	ricin B-like lectin R40G3	Defense signaling	Wheat - *Fusarium graminearum* (head blight)	Song et al., 2021
LOC115722789	59290403	59292733	F-box/FBD/LRR-repeat protein At3g26920-like	Potential R gene		
LOC115722965	59414491	59418457	aquaporin SIP1-2	ROS accumulation, defense signaling	Arabidopsis - *Pseudomonas syringae* (bacterial blight)	Tian et al., 2016

ROS, reactive oxygen species; SA, salicylic acid; PR1, pathogenesis related protein 1.

### 3.6 Microscopy analysis of PM2-mediated resistance response

**Figure 6:**
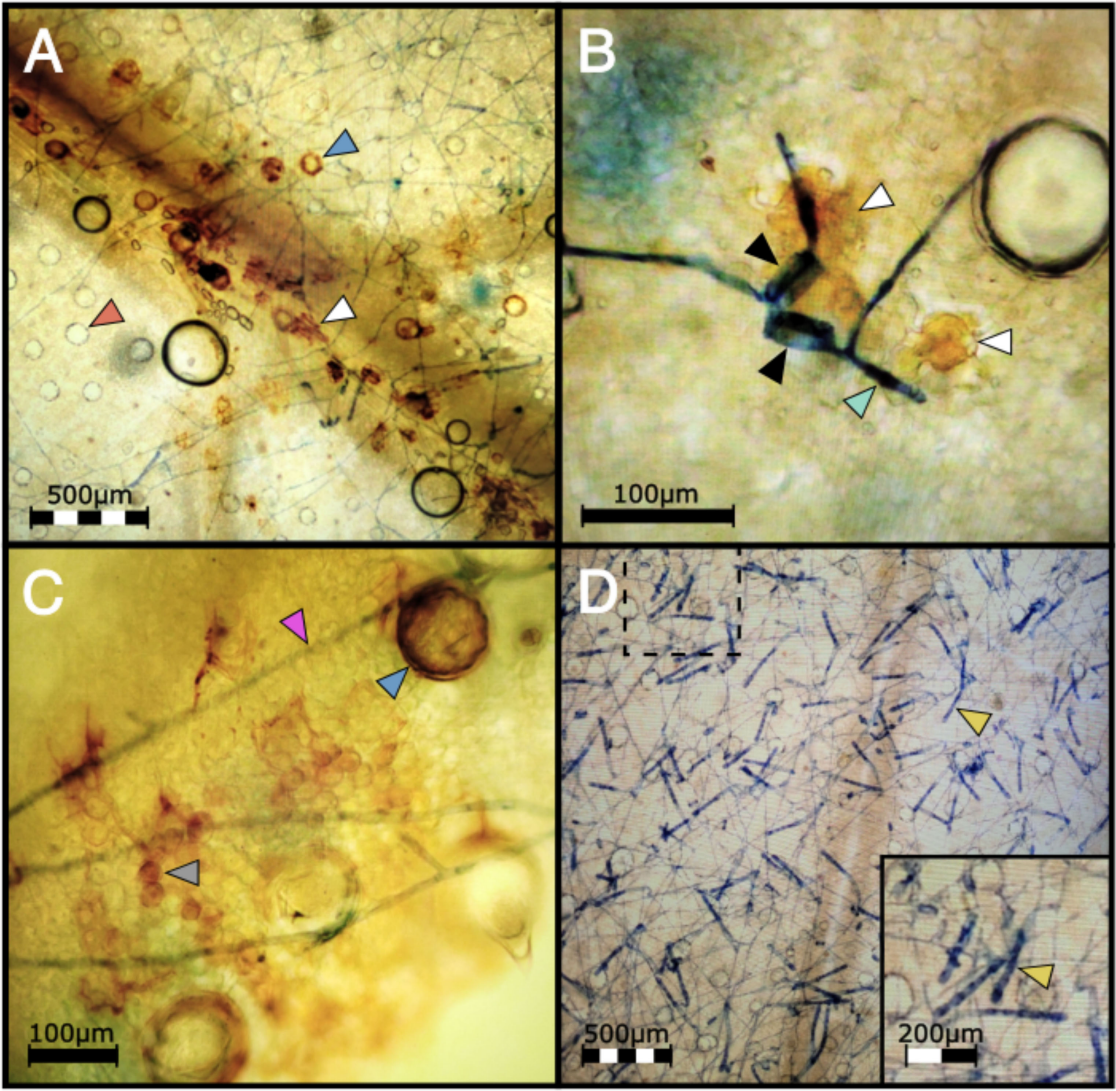
**(A)** DAB staining shows H_2_O_2_ accumulation in a highly localized pattern mainly in epidermal cells (white arrow) in PM2 genotype W03 infected with the mycelia of *G. ambrosiae* (string-like network stained in blue) at 1 wpi. Blue and red arrows show trichome basal cells with and without ROS accumulation respectively. **(B)** Epidermal cells (white arrows) showing ROS accumulation under two germinated PM spores (black arrows) with elongated germ tubes (light green arrow) attempting to penetrate the W03 host tissue. **(C)** Mesophyllic accumulation of H_2_O_2_ (round shape cells, grey arrow) was also observed in PM2 W03 samples under mycelial growth of *G. ambrosiae* (pink arrow). Blue arrow shows a trichome basal cell filled with ROS. **(D)** Susceptible genotype AC did not show ROS accumulation after DAB staining and showing high levels of conidiophore (yellow arrow) production instead. Dashed box denotes the magnified region in bottom right inset.

To investigate the defense mechanisms underlying PM2 resistance, DAB staining was employed to detect hydrogen peroxide (H_2_O_2_), a key indicator of the hypersensitive response in plants (Asselbergh et al., 2007; Seifi et al., 2013a). After infecting clones with PM pathogen, PM2-resistant genotypes showed strong and localized DAB staining in the epidermis and mesophyll cells of infected leaves (**Figures 6A–C**; **Supplementary Figure S2**) while no staining could be observed in the leaves of the susceptible genotype (**Figure 6D**).

**Figure 7:**
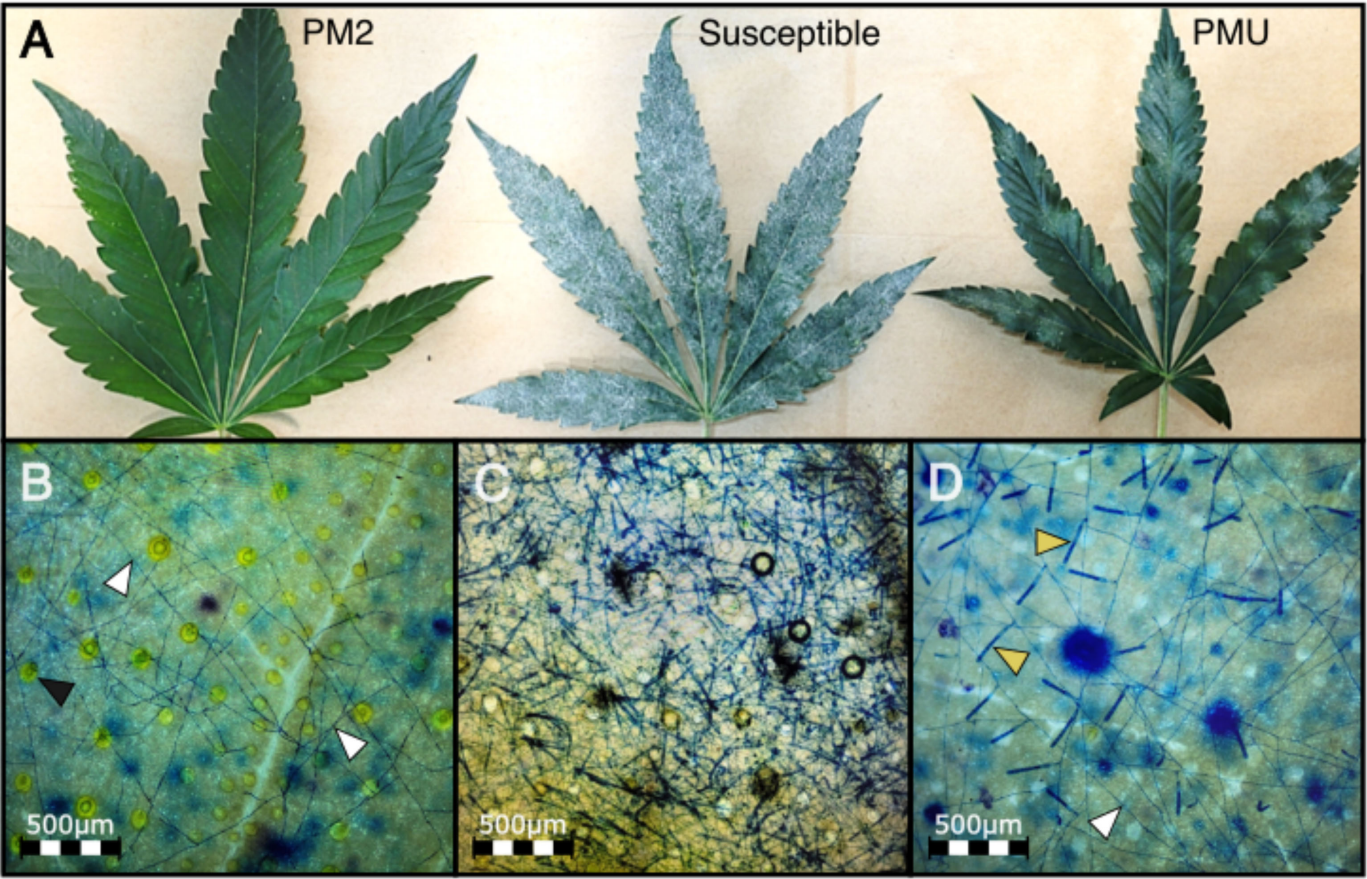
PMU and PM2-mediated response compared to a susceptible cannabis genotype at 4 wpi. **(A)** PM infected leaves from PM2 carrying genotype W03 (left), susceptible genotype AC (center), and PMU carrying genotype P04 (right). **(B)** Mycelial network (white arrows) developed on PM2 resistant genotype with no conidiophores. Black arrows denote basal trichome cells. **(C)** High density of conidiophore and sporulation can be seen on the susceptible genotype. **(D)** Low density of conidiophore (yellow arrows) generation can be seen in sporadic patches of PM mycelial growth (white arrow) on PMU.

In susceptible cannabis genotypes, the PM fungal pathogen *G. ambrosiae* fully develops its mycelial network and conidiophores within 2 wpi, completing its infectious life cycle. Disease development was compared between W03, AC, and a third genotype, P04, having an unknown form of PM resistance (PMU) identified in our clonal infection assay (**Figure 7A**). Clear differences in infection symptoms, mycelia growth, and pathogen reproduction were observed between PM2-mediated resistance and PMU. PM2-mediated resistance in W03 and N88 showed restricted mycelial growth and strong suppression of the conidiophore formation, which is a key developmental stage for the sporulation phase of the pathogen (**Figure 7B**, and **Supplementary Figure S2**). In contrast, susceptible genotype AC under identical conditions showed dense mycelial growth with high amounts of conidiophore formation (**Figure 7C**). While conidiophore formation and sporulation were largely suppressed in the PM2-mediated response, normal conidiophore formation and sporulation occurred in sporadic patches of restricted PM colonies in the PMU-induced response (**Figure 7D**). It should be noted that P04 scored a disease index value of 16.77 in our clonal infection assay and was therefore deemed resistant by our criteria (DI< 33). The reduced level of infection observed in W03 and N88 compared to P04 highlights the efficacy of PM2-mediated resistance.

**Figure 8:**
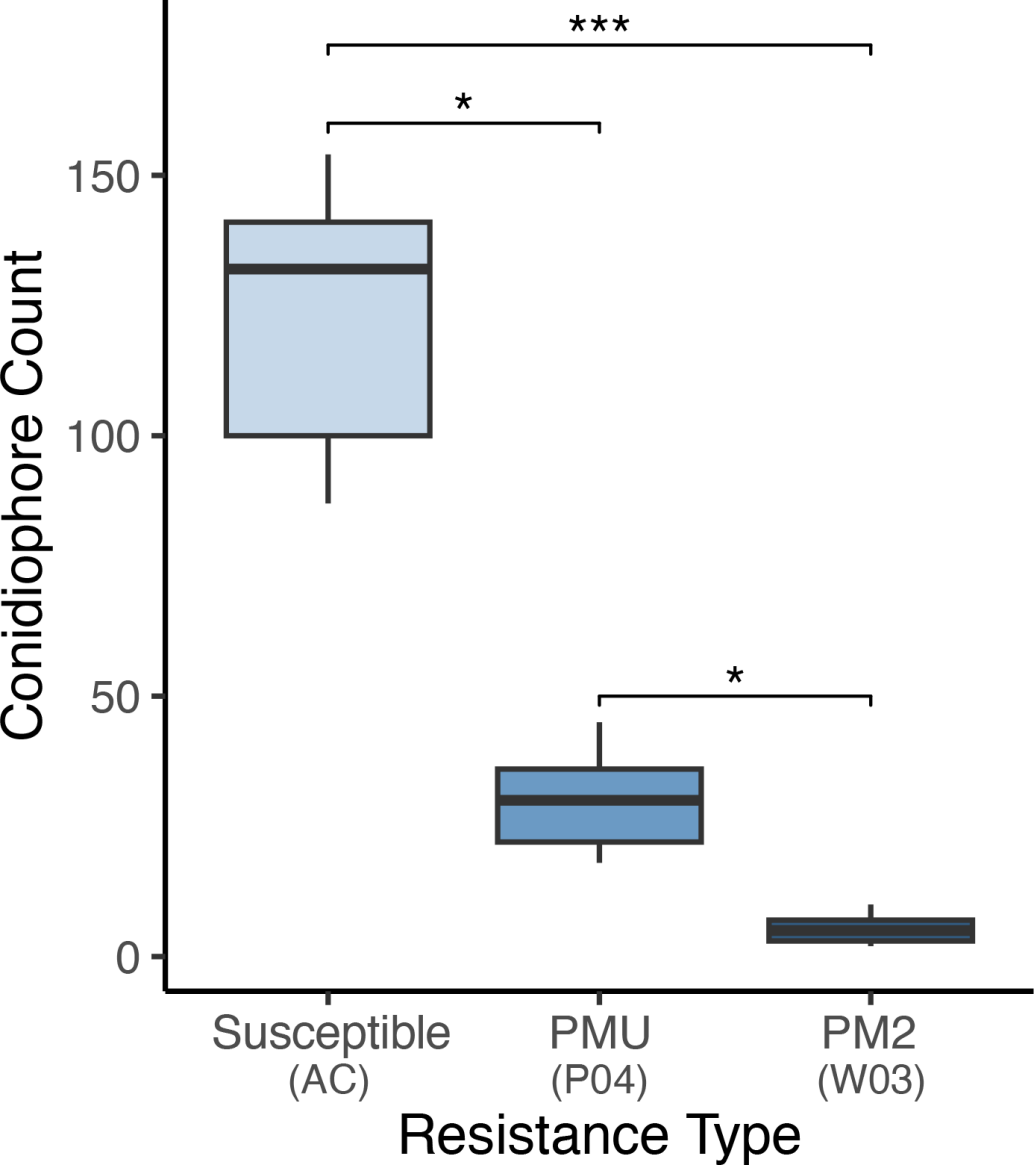
Powdery mildew conidiophore density observed on susceptible genotype AC, PMU resistant genotype P04, and PM2 resistant genotype W03. Values indicate mean number of conidiophores observed per 3.8 square mm of leaf surface. Conidiophores were counted in 6 plants per genotype in leaf samples collected from PM infected areas. replicates per genotype type. Asterisks denote level of significance between groups [Kruskal-Wallis test, *X* ^2^ (2, 27) = 23.15 with Dunn’s *post-hoc* test and Bonferroni correction (P*< 0.05, P***< 0.001)]. Centre line within boxes denote the median of the distribution, with box bottom and top lines indicating the first and third quartiles respectively. The upper and lower whiskers extending from boxes indicate the highest and lowest data points within each distribution respectively.

To validate the repression of conidiophore formation observed in PM2-mediated resistance, number of conidiophores per 3.8 mm^2^ of leaf surface were counted at 9 randomly selected positions on leaves of W03, P04, and susceptible genotype AC (**Figure 8**). Significant differences in mean conidiophore count were observed in all three genotypes [Kruskal-Wallis test, *X* ^2^ (2, 27) = 23.15, *p*< 0.001]. Mean conidiophore counts from W03 and P04 were significantly lower compared to susceptible AC, *p*< 0.001 and *p*< 0.05 respectively (Dunn’s *post-hoc* test with Bonferroni correction). When comparing PM2-mediated resistance to PMU, W03 had significantly lower counts of conidiophores compared to P04 (*p*< 0.05), with mean number of observed conidiophores at 5.33 compared to 35.44 for P04, and 118.33 for AC. These results further demonstrate the robust level of PM resistance conferred by PM2.

## 4 Discussion

Powdery mildew (PM) is the most prevalent fungal disease in indoor cannabis growing operations, causing significant losses to the industry (Pépin et al., 2021). Qualitative resistance to PM has been reported in hops (Humulus lupulus), a species closely related to cannabis (Henning et al., 2017). Recently, the first report of a single dominant resistance locus against PM was reported in cannabis, named PM1, with a suggested location of the causal R gene on chromosome 2 (CS10 Chr ID: NC_044375.1; GenBank acc. no. GCA_900626175.2) (Mihalyov and Garfinkel, 2021). In this study, using BSR-seq, we identified a new single dominant PM resistance locus, named PM2, located on chromosome 9 (CS10cChr ID: NC_044376.1). No macroscopic chlorotic spots were detected on PM2 leaves. However, DAB staining revealed H_2_O_2_ accumulation in epidermal and mesophyll cells beneath the pathogen’s mycelial growth, indicating a highly localized HR reaction in PM2-mediated resistance. Furthermore, the reproduction phase of the fungal pathogen was strongly suppressed, resulting in a significant reduction of conidia generation (>90%) in PM2 genotypes. This could be explained by the inhibition of penetration through HR by the resistant genotype, leading to the observed long ‘wandering’ mycelial growth with delayed and minimal reproduction. Such a phenomenon has been previously reported in an interaction between the biotrophic rust pathogen *Puccinia striiformis* and a resistant cultivar of wheat harboring the YR15 R gene (Seifi et al., 2021).

Further exploration focused on defense-associated genes flanking PM2 revealed several candidate genes (**Table 4**). Notably, these genes fall into three main functional categories: 1) hormonal regulation, particularly SA signaling pathway; 2) genes involved in ROS accumulation and cell death induction; and 3) genes predicted to encode potential R proteins, including one (LOC115722098) exhibiting LLR and Ser/Thr-kinase domains. Studies in other plant species have highlighted the role of annexin and aquaporin proteins in defense responses against biotrophic pathogens. For example, annexin proteins regulate SA-dependent defense responses and influence ROS generation and callose deposition (Shi et al., 2023), while aquaporin proteins link extracellular ROS accumulation to intracellular defense signaling in Arabidopsis challenged by pathogens (Tian et al., 2016). *RPP2B*, an R gene with a TIR-NB-LRR domain, plays a crucial role in race-specific recognition and defense signaling against downy mildew (Sinapidou et al., 2004). Similarly, the TIR-NB-LRR DSC1 protein is implicated in basal immunity against root-knot nematodes in Arabidopsis (Warmerdam et al., 2020). Research on the TCA cycle through glutamate dehydrogenase suggests its role in defense responses against biotrophic pathogens, including the induction of programmed cell death (Seifi et al., 2013b). Accelerated Cell Death 6 (ACD6), involved in SA-mediated defense responses in Arabidopsis, triggers programmed cell death and expression of PR genes against bacterial pathogens (Dong, 2004). Notably, ACD6 belongs to the transmembrane-ankyrin-repeat protein family with a key regulating role in tradeoffs between vegetative growth and general pathogen defense, conferring broad-spectrum disease resistance to pathogens including PM in Arabidopsis (Todesco et al., 2010). Syntaxin proteins have been identified as crucial players in plant defense responses to PM. Studies on barley and Arabidopsis suggest that syntaxin genes regulate fungal penetration and enhance resistance against PM pathogens (Bracuto et al., 2017). Notably, silencing of the syntaxin gene SlPEN compromised resistance in a *mlo*-resistant tomato line against *Oidium neolycopersici*, highlighting their importance in PM resistance (Bracuto et al., 2017).

Originally developed using restriction fragment length polymorphisms (RFLPs) and random amplified polymorphic DNAs (RAPDs) markers (Michelmore et al., 1991), BSA now employs the use of next-generation sequencing (NGS) technologies which have greatly improved its power of detection in mapping phenotypic traits. Algorithms designed for detecting marker associations to target traits have been developed for NGS-based BSA (also referred to as BSA-Seq). These algorithms are primarily based on detecting differences in allele frequencies between bulks, paired with smoothing techniques that help identify positive signals from noise. Popular BSA-Seq statistics include the G’ value (Magwene et al., 2011), SNP-index/InDel-index (Takagi et al., 2013; Singh et al., 2017), Euclidean distance (ED, Hill et al., 2013), and smoothLOD (Zhang et al., 2019), among others (see Li and Xu, 2022). In addition to whole genome resequencing, bulked segregant analysis using SNP markers called from transcriptome data (BSR-Seq) has been successfully used to map QTLs in maize (Liu et al., 2012), zebrafish (Hill et al., 2013), pacific white shrimp (Dai et al., 2018), pea (Wu et al., 2022) and wheat (Trick et al., 2012; Li et al., 2018; Hao et al., 2019). Several BSA statistics have been developed specifically to handle the challenges inherent to RNA-Seq data, such as allele-specific expression and variable coverage. These include a Bayesian approach to estimate marker association (Liu et al., 2012) and the ED metric (Hill et al., 2013), both of which were successfully used in the present study to map PM2 mediated resistance in cannabis. Both the Bayesian and Euclidean distance methods identified the same 2Mb region within chromosome 9, containing the PM2 QTL. While the Bayesian method required considerably longer computational times, the signal to noise ratio was superior to ED, leaving an easily identified signal peak associated with PM resistance. The comparison of two mapping populations segregating for PM2 provided further strength to our analysis. Both populations showed clear signals for PM2 mediated resistance in the same location on chromosome 9. A minor secondary peak was observed on chromosome 7, however this was not investigated in this study as it was observed in the W03xAC F_1_ population only.

The use of RNA-Seq data for SNP calling provides the added benefit of gene expression data from both resistant and susceptible bulks. Several gene candidates for PM2 mediated resistance identified by BSR-Seq showed increased gene expression in resistant bulks compared to susceptible bulks, including *ACD6*, *Annexin D8*, *RPP2B*, and *DSC1*. This gene expression data, however, comes from pooled samples with no biological replicates, and therefore should not be used to draw conclusions, but to guide follow-up studies.

Populations frequently used in BSA include bi-parental populations segregating for a trait of interest. Common types include F_2_, F_2:3_, BC_1_, and recombinant inbred lines (Zou et al., 2016). However, any population containing individuals with contrasting traits can in theory be used in a bulked sample analysis (Li and Xu, 2022). For species that have long life cycles, or are not amenable to selfing, F_1_ populations have been used (Shen et al., 2019; Dougherty et al., 2018). These populations require parents to be highly heterozygous and have observable phenotypic variation in the F_1_ generation, as is the case with dominant traits (Li and Xu, 2022). Both our N88xAC and W03xAC F_1_ populations exhibited clear segregation in PM resistance; employing these populations saved a considerable amount of time, by removing the need to proceed to the F_2_ generation.

The development of resistant cultivars is an effective practice for the management of PM disease control. Successful introgression of PM2 mediated resistance into elite cannabis cultivars will reduce the reliance of chemical pesticides, which are heavily regulated in cannabis as in most other crops. As the cannabis industry expands by developing new markets and applications, there will be an increased need for resistant cannabis cultivars, highlighting the relevance of studies, such as the one presented here, identifying the genetic basis of resistance and susceptibility to pathogens in this species.

## Data Availability

RNA-Seq data used for BSR-Seq in both N88xAC and W03xAC F1 populations has been uploaded to NCBI’s sequence archive under BioProject ID PRJNA1182366.
