# Supplementary material for: Mapping and characterization of a novel powdery mildew resistance locus (PM2) in Cannabis sativa L
Source: Front Plant Sci. 2025 Mar 13;16:1543229. doi: 10.3389/fpls.2025.1543229 (PMC11966446; doi:10.3389/fpls.2025.1543229)
Supplement: Supplementary file 1 [file DataSheet1.docx]

**Mapping and characterization of a novel powdery mildew resistance locus (PM2) in *Cannabis sativa* L.**

Soren Seifi^1^†, Keegan M. Leckie^1^†, Ingrid Giles^1^, Taylor O’Brien^1^, John O. MacKenzie^1^, Marco Todesco^2^, Loren H. Rieseberg^2^, Gregory J. Baute^3^, Jose M. Celedon^1^*

*Corresponding author

† These authors contributed equally to this work and share first authorship

**Supplemental Material**

**Supplemental Figure S1.** Mapping the PM2 locus using BSA in the CS10 CBDRx reference genome.

**Supplemental Figure S2.** DAB staining and H_2_O_2_ accumulation in N88.

**Supplemental Table S1.** PACE Sequence primers used for genetic markers tracking PM2 resistance.

**Supplemental Table S2.** Genetic markers associated with PM2 resistance and their location in reference genomes.

**Supplemental Table S3.** RNA-Seq gene expression data for genes within the PM2 locus for both W03xAC and N88xAC F1 bulks.

**
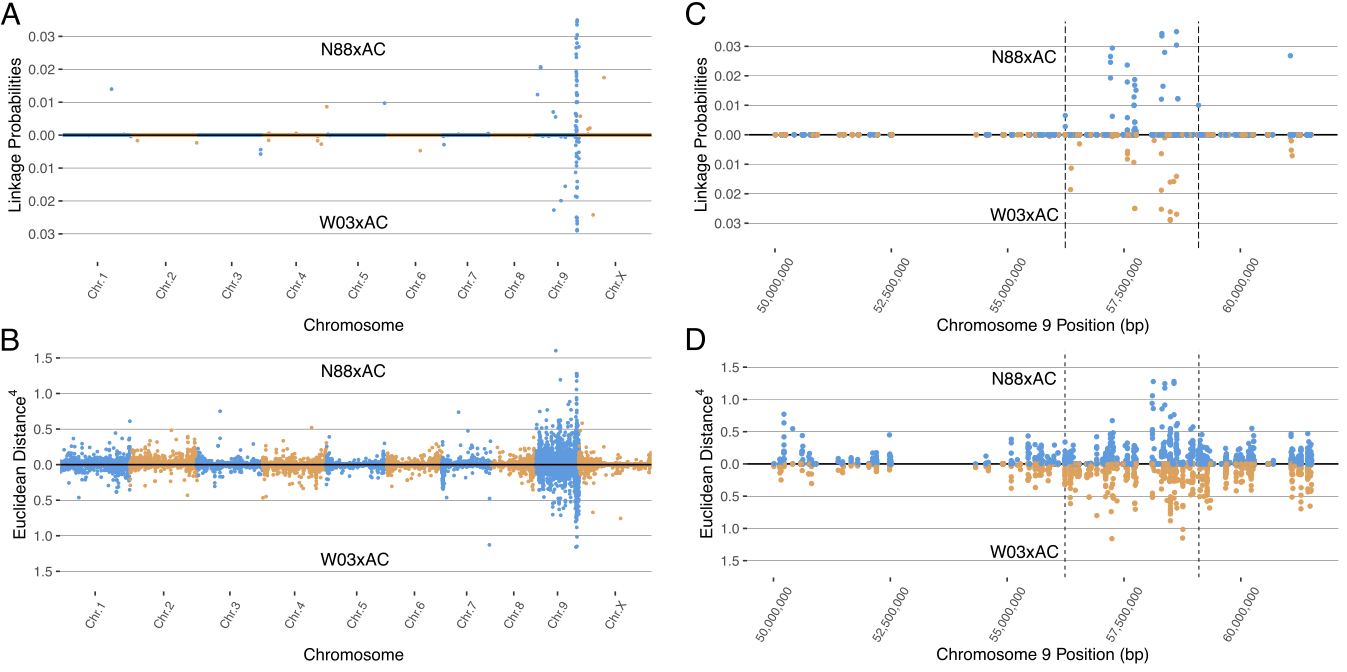
**

**Supplemental Figure S1.** Mapping the PM2 locus using BSA in both N88xAC (top half) and W03xAC (bottom half) F1 segregating populations. CBDRx (GenBank ass. no. GCA_900626175.2) genome was used as reference. **(A)** BSA mapping using the Bayesian implementation to estimate the probability of SNPs linked to PM2 resistance. **(B)** BSA mapping using the Euclidean distance (ED) metric. ED values for each SNP have been raised to the 4^th^ power to increase signal to noise ratio. Alternating colours denote chromosomes in **(A)** and **(B)**. Both Bayesian **(C)** and ED **(D)** metrics identify a region on chromosome 9 between 56,237,869 and 59,102,577 containing SNPs associated with PM2 resistance. Vertical dashed lines denote the boundaries of the PM2 associated region, defined by increased ED scores. Blue colour denotes values for N88xAC while orange denotes values for W03xAC in **(C)** and **(D)**.


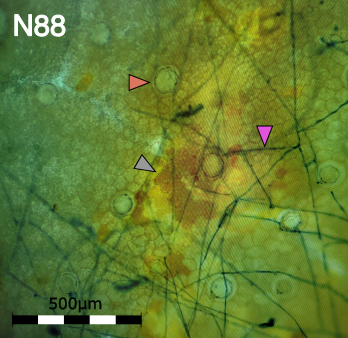


**Supplemental Figure S2.** DAB staining showing H_2_O_2_ accumulation in mesophyllic cells (grey arrow) in PM2 genotype N88. Pink arrow denotes PM mycelium (string-like network stained blue) growing on the leaf surface. Red arrow shows trichome basal cell.

**Supplemental Table S1**. PACE sequence primers used for genetic markers tracking PM2 resistance.

| **Marker** | **Forward Primer 1** | **Forward Primer 2** | **Common Reverse Primer** |
| --- | --- | --- | --- |
| MR110 | CACTTTCTTCAAGTCATCCT  CACTC | CCACTTTCTTCAAGTCATCC  TCACTT | CGACSGATATGTTCTTTTCG  GGGAA |
| MR121 | GGCCATTGCTAGATAATTCC  GGT | GGCCATTGCTAGATAATTCC  GGG | GTGCATGGCAGAGGATCACA  CATTT |
| MR124 | TGGAAAGAGAAGAARAATGA  AGCAGAA | GGAAAGAGAAGAARAATGAA  GCAGAC | GTTCCTGAAAAACGGAGTTG  ATTCAGTTT |
| MR125 | TTACTTGGTCAACCTGGAAC  AGTC | TTTACTTGGTCAACCTGGAA  CAGTT | ACCCARTCCAAGATCAACAA  GATATAGTTT |
| MR131 | TTTGKGTTGGAAAGAGAAGA  AG | CTTTTGKGTTGGAAAGAGAA  GAAA | GTTCCTGAAAAACGGAGTTG  ATTCAGTTT |

**Supplemental Table S2**. Genetic markers associated with PM2 resistance and their location in reference genomes.

| **Marker** | **Ref** | **Alt** | **Pink Pepper** | | **CBDRx** | |
| --- | --- | --- | --- | --- | --- | --- |
|  |  |  | **Chromosome** | **Position** | **Chromosome** | **Position** |
| MR110 | C | T | NC_083609.1 | 58,448,827 | NC_044376.1 | 58,108,802 |
| MR121 | G | T | NC_083609.1 | 58,552,298 | NC_044376.1 | 58,570,897 |
| MR124 | A | C | NC_083609.1 | 58,289,703 | NC_044376.1 | 58,423,201 |
| MR125 | G | A | NC_083609.1 | 58,164,119 | NC_044376.1 | 58,314,046 |
| MR131 | G | A | NC_083609.1 | 58,289,715 | NC_044376.1 | 58,423,213 |

Notes: Chromosome coordinates of markers are provided in both Pink Pepper and CBDRx reference genomes. Ref indicates reference allele found in the Pink Pepper reference genome and Alt the alternative allele.

**Supplemental Table S3.** Provided as a standalone excel file “Supplemental Table S3.xlsx”.
